# Supplementary material for: A Single Center Observational Study on Clinical Manifestations and Associated Factors of Pediatric Long COVID
Source: Int J Environ Res Public Health. 2023 Sep 21;20(18):6799. doi: 10.3390/ijerph20186799 (PMC10531477; doi:10.3390/ijerph20186799)
Supplement: Supplementary file 1 [file ijerph-20-06799-s001.zip › ijerph-2581798-supplementary.pdf]

Department of Maternal Infantile and Urological Sciences
"SAPIENZA" UNIVERSITY OF ROME
Viale Regina Elena, 324 – 00161 Roma

Date___________________

Surname ______________________ Name ______________________

Telephone number_____________________e-mail address____________________

Gender_______________________________

Date of birth __________________________

Nasopharyngeal swab positive for SARS-CoV-2 (date)________________

Nasopharyngeal swab negative for SARS-CoV-2 (date)________________

Perinatal factors

Gestational age (weeks)	________
Preterm (<37 wk)	YES NO
Spontaneous delivery	YES NO
Cesarean delivery	YES NO
Breastfeeding (at least 3 months after birth)	YES NO
**Family history**	
Maternal asthma	YES NO
Paternal asthma	YES NO
Sibling wheezing/asthma	YES NO
Maternal eczema	YES NO
Paternal eczema	YES NO
Sibling eczema	YES NO
**Environmental factors**	
Smoking in parental house	YES NO
**Past history**	
Previous bronchiolitis	YES NO
Previous wheezing	YES NO
Atopy skin prick test	YES NO
**Does the child have chronic diseases?**	YES NO

If the child has chronic diseases, please specify:____

Did the child have any of the following general symptoms during SARS-CoV-2 infection?

Fever (temperature of 37.5° C or greater) YES NO

Respiratory Symptoms

Cough (wet or dry) YES NO

Dyspnoea (difficulty breathing or short of breath) YES NO

Rhinitis (nasal congestion, runny nose) YES NO

Pharyngitis (sore throat) YES NO

Otitis (middle ear infection/inflammation with swelling and redness) YES NO

Exercise induced dyspnea YES NO

Other respiratory symptoms______________________________

Gastrointestinal Symptoms

Vomiting YES NO

Abdominal pain YES NO

Stomach ache YES NO

Poor Appetite (not feeling hungry, eating much less than usual, or not eating at all) YES NO

Increase in food intake (excessive hunger and desire for food) YES NO

Other gastrointestinal symptoms______________________________

Neurological Symptoms

Headache (pain in any region of the head that lasts at least 15 minutes) YES NO

Anosmia (a sudden loss of sense of smell) YES NO

Ageusia (a sudden loss of sense of taste) YES NO

Altered taste YES NO

Altered Smell YES NO

Lack of Concentration (unable to think clearly, focus on a task, or maintain attention) YES NO

Loss of memory (unusual forgetfulness) YES NO

Sleeping disorders (such as insomnia, sleepwalking, sleep talking, nightmares) YES NO

Anxiety (fear, worry or lack confidence to try new things) YES NO

Syncope (temporary loss of consciousness) YES NO

Dizziness (vertigo) YES NO

Other neurological symptoms______________________________

Other symptoms (please specify)______________________________

Did the child need hospital admission or drugs during the acute phase of the disease? YES NO

If yes, please specify______________________________

Did the child have any of the following general COVID-19 related symptoms 30 days after the SARS-CoV-2 infection ?

Fever (defined as having a temperature of 37.5° C or greater) YES NO

Respiratory Symptoms

Cough (wet or dry) YES NO
Dyspnoea (difficulty breathing or short of breath) YES NO
Rhinitis (nasal congestion, runny nose) YES NO
Pharyngitis (sore throat) YES NO
Otitis (middle ear infection/inflammation with swelling and redness) YES NO
Exercise induced dyspnea YES NO
Other respiratory symptoms____

Gastrointestinal Symptoms

Vomiting YES NO
Abdominal pain YES NO
Stomach ache YES NO
Poor Appetite (not feeling hungry, eating much less than usual, or not eating at all) YES NO
Increase in food intake (excessive hunger and desire for food) YES NO
Other gastrointestinal symptoms____

Neurological Symptoms

Headache (pain in any region of the head that lasts at least 15 minutes) YES NO
Anosmia (a sudden loss of sense of smell) YES NO
Ageusia (a sudden loss of sense of taste) YES NO
Altered taste YES NO
Altered Smell YES NO
Lack of Concentration (unable to think clearly, focus on a task, or maintain attention) YES NO
Loss of memory (unusual forgetfulness) YES NO
Sleeping disorders (such as insomnia, sleepwalking, sleep talking, nightmares) YES NO
Anxiety (fear, worry or lack confidence to try new things) YES NO
Syncope (temporary loss of consciousness) YES NO
Dizziness (vertigo) YES NO
Other neurological symptoms____

Other symptoms (please specify)____

Did the child need hospital admission or drugs (COVID-19 related) 30 days after the SARS-CoV-2 infection?
YES NO

If yes, please specify____

Weight (Kg)____________ Height (cm)___________BMI____________

General physical examination

____

____

____

____

____

____

PHONE QUESTIONNAIRE 90 DAYS AFTER THE INFECTION

Did the child have any of the following general COVID-19 related symptoms 90 days after the SARS-CoV-2 infection?

Fever (temperature of 37.5° C or greater) YES NO

Respiratory Symptoms

Cough (wet or dry) YES NO
Dyspnoea (difficulty breathing or short of breath) YES NO
Rhinitis (nasal congestion, runny nose) YES NO
Pharyngitis (sore throat) YES NO
Otitis (middle ear infection/inflammation with swelling and redness) YES NO
Exercise induced dyspnea YES NO
Other respiratory symptoms____________________

Gastrointestinal Symptoms

Vomiting YES NO
Abdominal pain YES NO
Stomach ache YES NO
Poor Appetite (not feeling hungry, eating much less than usual, or not eating at all) YES NO
Increase in food intake (excessive hunger and desire for food) YES NO
Other gastrointestinal symptoms____________________

Neurological Symptoms

Headache (pain in any region of the head that lasts at least 15 minutes) YES NO
Anosmia (a sudden loss of sense of smell) YES NO
Ageusia (a sudden loss of sense of taste) YES NO
Altered taste YES NO
Altered Smell YES NO
Lack of Concentration (unable to think clearly, focus on a task, or maintain attention) YES NO
Loss of memory (unusual forgetfulness) YES NO
Sleeping disorders (such as insomnia, sleepwalking, sleep talking, nightmares) YES NO
Anxiety (fear, worry or lack confidence to try new things) YES NO
Syncope (temporary loss of consciousness) YES NO
Dizziness (vertigo) YES NO
Other neurological symptoms____________________

Other symptoms (please specify)____________________

Did the child need hospital admission or drugs (COVID-19 related) 90 days after the SARS-CoV-2 infection ? YES NO

If yes, please specify____________________
